# Combining denoising of RNA-seq data and flux balance analysis for cluster analysis of single cells

**DOI:** 10.1186/s12859-022-04967-6

**Published:** 2022-10-25

**Authors:** Bruno G. Galuzzi, Marco Vanoni, Chiara Damiani

**Affiliations:** 1 Department of Biotechnologies and Biosciences, University of Milano-Bicocca, Milan, Italy; 2 SYSBIO Centre of Systems Biology/ ISBE.IT, Milan, Italy

**Keywords:** Metabolic networks, scRNA-seq, Flux balance analysis

## Abstract

**Background:**

Sophisticated methods to properly pre-process and analyze the increasing collection of single-cell RNA sequencing (scRNA-seq) data are increasingly being developed. On the contrary, the best practices to integrate these data into metabolic networks, aiming at describing metabolic phenotypes within a heterogeneous cell population, have been poorly investigated. In this regard, a critical factor is the presence of false zero values in reactions essential for a fundamental metabolic function, such as biomass or energy production. Here, we investigate the role of denoising strategies in mitigating this problem.

**Methods:**

We applied state-of-the-art denoising strategies - namely MAGIC, ENHANCE, and SAVER - on three public scRNA-seq datasets. We then associated a metabolic flux distribution with every single cell by embedding its noise-free transcriptomics profile in the constraints of the optimization of a core metabolic model. Finally, we used the obtained single-cell optimal metabolic fluxes as features for cluster analysis. We compared the results obtained with different techniques, and with or without the use of denoising. We also investigated the possibility of applying denoising directly on the Reaction Activity Scores, which are metabolic features extracted from the read counts, rather than on the read counts.

**Results:**

We show that denoising of transcriptomics data improves the clustering of single cells. We also illustrate that denoising restores important metabolic properties, such as the correlation between cell cycle phase and biomass accumulation, and between the RAS scores of reactions belonging to the same metabolic pathway. We show that MAGIC performs better than ENHANCE and SAVER, and that, denoising applied directly on the RAS matrix could be an effective alternative in removing false zero values from essential metabolic reactions.

**Conclusions:**

Our results indicate that including denoising as a pre-processing operation represents a milestone to integrate scRNA-seq data into Flux Balance Analysis simulations and to perform single-cell cluster analysis with a focus on metabolic phenotypes.

## Scientific background

Metabolism generates energy from food supply, provides building blocks for macromolecular biosynthesis, and plays a pivotal role within cellular functions. It is regulated by a sophisticated web of interactions that respond to the overall physio-pathological state. In turn, several metabolites control signal transduction and epigenetic pathways. As a result, metabolism is in a privileged position to integrate metabolic, genetic, epigenetic, and environmental signals, so that each physio-pathological condition can be associated with a specific metabolic state [1, 2].

Metabolic networks represent powerful instruments to study metabolism and cell physiology (e.g., growth rate, ATP production, and metabolic fluxes in general) under different conditions [3, 4]. Such networks are generally studied with Constrained-Based Reconstruction and Analysis (COBRA) methods, with the aim of simulating numerically metabolic fluxes, that is, the net velocity of the biochemical reactions. Indeed, experimental determination of metabolic fluxes is currently not high-throughput, especially at the single-cell level. Therefore, single-cell metabolic fluxes must be estimated numerically from the integration of other single-cell omics data (e.g. RNA-seq) into constrained-based models.

The starting point of COBRA modeling is the information embedded in the metabolic network, which can be represented with a stoichiometric matrix *S* of dimension $M\times R$, where *M* is the number of metabolites and *R* is the number of reactions. In a COBRA model, a steady-state condition is imposed, that is, the total production of any metabolite must equal the total amount of its consumption. Hence, any possible metabolic flux configuration is represented by a vector $\vec {v}$, for which $S \vec {v}=0$, i.e. the null space of the stoichiometric matrix. Because of the large number of possible feasible flux distributions, different strategies have been implemented to predict the target metabolic flux distribution. The COBRA method par excellence is Flux Balance Analysis (FBA) [5], which calculates a single feasible flux distribution that maximizes/minimizes the flux through a target objective function, e.g. the biomass or ATP production. An important application of COBRA models consists in analyzing how variations in mRNA levels impact on metabolic phenotypes, that is, on the metabolic fluxes computed through FBA, as reviewed in [6–8]. In a nutshell, the goal is to incorporate the specific transcriptomics profile of a biological sample, either bulk or single-cell, into the objective function or in the constraints of the FBA problem. The optimal flux distributions, one for each different transcriptomics profile, can represent a set of new features that can be used to cluster the biological samples [9, 10].

Incorporation of the gene expression data into the objective function is most typically used when the aim is to reconstruct a tissue-specific metabolic network. For example, the INIT algorithm seeks for a flux distribution that maximizes the flux through highly expressed reactions while minimizing the flux through lowly expressed reactions [11, 12]. The resulting active sub-network represents the draft of the tissue-specific network.

Incorporation of gene expression data into flux constraints is instead more typically used when the aim is to obtain a plausible flux distribution of the system under study, without necessarily delivering a specific model. This is the case when one wants to characterize the flux distribution of single-cells within a mixed cell population.

This latter kind of integration is generally based on Gene Protein Reaction (GPR) associations, which describe the relationship between genes and reactions and among genes possibly associated with the same reaction. More in detail, the GPRs represent logical formulas describing how gene products concur to catalyze a given reaction, using Boolean operators. The AND operator is used when distinct genes encode different sub-units of the same enzyme, whereas the OR operator is used for genes encoding isoforms of the same enzyme. Starting from the mRNA abundances of the genes partaking in a specific reaction, current approaches evaluate the GPR of the reaction by using either Boolean [13], or continuous approaches [14, 15]. In the former scenario, the flux of a given reaction is constrained to zero if any essential gene (i.e., joined with an AND operator) has a zero expression value (or it is lower than a given threshold), it is left unbound otherwise [13]. In the latter scenario, that we address in this work, a continuous Reaction Activity Score (RAS) is obtained by using a specific operation for genes joined by the AND or by the OR operator. Generally, the sum of the RNA levels [9] or the maximum RNA level [16] is considered for OR-joined genes, whereas the minimum [9] or mean level is considered for AND-joined genes. The sets of RAS values, one for each metabolic reaction equipped with a GPR, is then used to derive specific upper bounds for the fluxes [9, 10, 15, 17].

The integration of bulk transcriptomics data into metabolic networks based on RAS-derived constraints has been widely explored, however only a few works [14, 15] have studied the integration of scRNA-seq data, which requires special attention as they are more subject to noise and false zero values. In fact, the high number of zeros in the scRNA-seq count matrix can result in a high number of fluxes constrained to be zero, thus possibly causing feasibility problems of FBA solutions.

In [15], the problem of false zero values was handled by comparison with bulk data. If a gene had a non-zero read count in the bulk, but a zero read count in each single-cell, the zero values were replaced with the bulk value. If, after this correction, the flux of some reaction that is essential to achieve an expected metabolic function was still constrained to zero, then the zero-constraint was replaced with a small arbitrary value. However, corresponding bulk data are not always available for scRNA-seq datasets.

Wagner et al. [14] used a different approach. They smoothed the read counts to mitigate the sparseness and stochastic of single-cell measurements, but they used the RAS values to derive an objective function, thus avoiding *in toto* feasibility problems due to constraining essential reactions to zero. Moreover, they did not focus on unsupervised clustering of single cells, but they performed differential reaction expression analysis between pathogenic and non-pathogenic Th17 cells.

Although the use of denoising strategies to improve cluster analysis of scRNA-seq datasets has been widely explored and debated [18, 19], to the best of our knowledge, no specific study has been made so far to assess their importance when integrating scRNA-seq data into a metabolic network.

In this work, we explored the possibility of applying denoising strategies as a systematic way to remove false zero RAS values and to obtain a more realistic clustering of single-cell flux distributions. To this aim, we selected three public scRNA-seq datasets, for which prior information about the expected clustering was available. We studied the effect of applying denoising either on the read counts matrix or after the RAS computation (i.e., on the RAS matrix). Finally, we compared the results of three different state-of-the-art denoising methods, which represent the best compromise between accuracy, usability and efficiency, according to [18], namely MAGIC, ENHANCE, and SAVER.

## Results

### Denoising reduces the level of sparsity of the RAS matrix

We computed the RAS values from the normalized read counts (TPM) for the three selected datasets using the ENGRO2 metabolic network model. For each dataset, we retained only the metabolic genes included in the ENGRO2 model (i.e., 494 genes). Note that not all the genes in the model have an expression value in the TPM matrix. Indeed, there is a percentage of coverage that varies between $72.87\%$ (GSE110949) and $92.71\%$ (E-GEOD-86618). At the end of the RAS computation, we obtained a RAS matrix where the columns represent the cells, and the rows represent the number of metabolic reactions for which at least one of the genes included in its GPRs was present in the read counts matrix. Such number of reactions varies between 302 ($89.6\%$ of model reactions associated with a GPR) for the GSE110949 dataset and 330 (97.92%) for the E-GEOD-86618 dataset.

**Table 1:** Levels of sparsity of read counts matrix, metabolic read counts matrix, RAS matrix, and RAS matrix restricted to reactions essential for biomass synthesis rate

Dataset	Count matrix (%)	Metabolic count matrix (%)	RAS matrix (%)	Essential RAS matrix (%)
GSE110949	80.70	60.06	62.87	55.64
E-GEOD-86618	56.18	28.35	25.01	13.47
GSE118056	81.46	60.89	59.91	51.43

We investigated the level of sparsity of RAS matrices. Table 1 reports the level of sparsity of the read counts matrix, the metabolic count matrix (i.e., the read counts matrix restricted to the metabolic genes, only), the RAS matrix, and the essential RAS matrix (i.e., the RAS matrix restricted to the reactions essential for biomass synthesis). In general, the level of sparsity of the RAS matrix and the metabolic count matrix result quite similar and lower than the ones of the original count matrices. Such similarity is due to the fact that most of the GPRs are made of a single gene only.

**Fig. 1:**
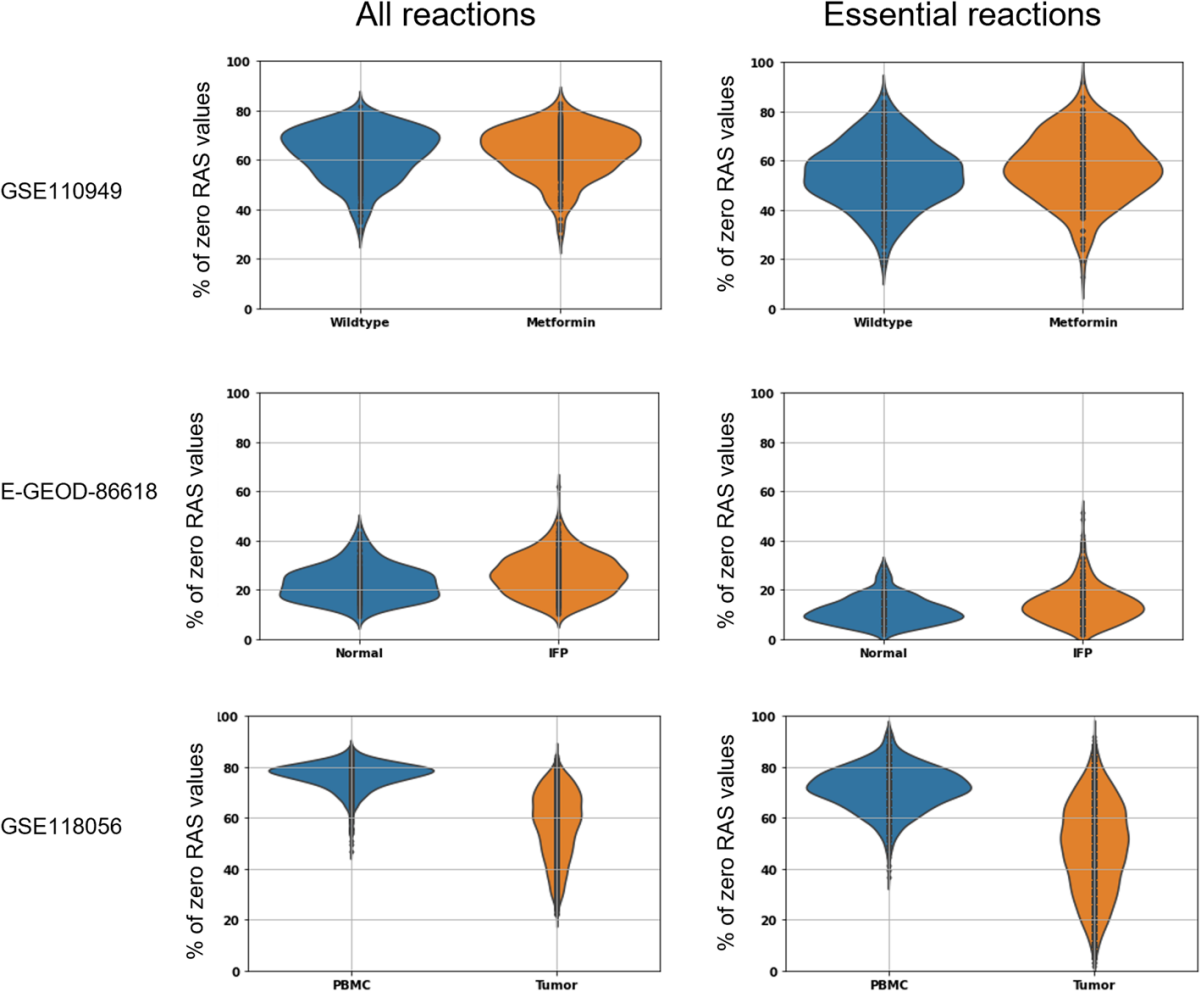
Sparsity analysis on the RAS matrices. Violin plots showing the distribution of the cells on the base of the percentage of zero RAS values for all reactions (left) and of the essential reactions for the biomass synthesis (right). We grouped the cells on the base of *a priori* knowledge specific of each dataset

To investigate the distribution of the zeros across the cells, we reported in Fig. 1 (left) the distribution of the cells on the base of the percentage of zero RAS values for all the reactions. We can note that, without using any denoising correction, such datasets present a high number of cells having a high percentage of zero values. In particular, in dataset GSE110949, most cells have more than $60\%$ of their RASs that is zero, without any significant difference between wild-type and metformin-adapted. In the dataset E-GEOD-86618, the level of sparsity is lower, with most cell having $20\%$ of their RASs that is zero, and without any significant difference between normal and IFP cells. The lower level of sparsity of this dataset is due to the lower level of sparsity of the read count matrix for the subset of metabolic genes, which is around $28\%$. Finally, in dataset GSE110949, most cells have more than $60\%$ of their RASs that is zero, with a strong difference between PBMC and cancer cells. Such a difference is also present in the distribution of zero-value read counts between PBMC and tumor cell. Worth of note, not only the mean number of zero values of the two cell populations is different (74% for PBMC and 67% for tumor cells), but also the variability in the cancer cell sub-population is much higher. Although PMBC can further differentiate [20], a larger heterogeneity in cancer cells is not unexpected, given the known ability of cancer cells to de-differentiate [21].

Such a high number of zeros RAS values could represent a problem when one wants to use them to constrain the upper bounds of the associated fluxes in the metabolic network, as per Eqs. 7 and 8, with $\epsilon =0$. Indeed, considering the FBA problem in Eq. 2, if a cell presents a zero RAS value in any essential reaction for $v_i$, then we obtain $v_i=0$ for that cell.

To analyze more in detail such problem, in Fig. 1 (right), we reported the distribution of the cells on the base of the percentage of zero RAS values, limited to the set of reactions essential for biomass synthesis. The biomass synthesis rate is a typical target objective function, which describes the composition of metabolites that make up the cell. For this objective, ENGRO2 has 82 essential reactions for biomass synthesis. Therefore, if a flux associated with any essential reaction is off (i.e., 0), then we will have no biomass synthesis rate. It is evident in Fig. 1 (right) that almost all cells from all datasets have a zero RAS value for at least one reaction essential for biomass synthesis. Therefore, performing FBA, using constrains of Eqs. 7 and 8 with biomass synthesis rate as objective target, would not produce growth for any cell. However, such fact appears to be unrealistic, especially for the first and the third dataset, in view of the fact that cancer cells are proliferative.

**Table 2:** Levels of sparsity of the RAS matrix after denoising application on the read counts matrix or on the RAS matrix

	Denoising on read counts matrix			Denoising on RAS matrix		
Dataset	MAGIC	ENHANCE	SAVER	MAGIC	ENHANCE	SAVER
GSE110949	0.05% (0%)	8.27% (4.36%)	0% (0%)	1.04% (0%)	10.59% (5.56%)	0.99% (0%)
E-GEOD-86618	0.01% (0%)	4.77% (2.47%)	0% (0%)	0.04% (0%)	3.63% (1.23%)	0% (0%)
GSE118056	1.91% (0.01%)	5.58% (1.74%)	0% (0%)	1.07% (0%)	9.68% (5.37%)	0% (0%)

We reported in the brackets the level of sparsity for the essential reactions for biomass synthesis rate

We evaluated the effect of applying three denoising strategies on the three RAS matrices, namely MAGIC, ENHANCE, and SAVER. At the first instance, we applied such strategies to the read counts matrix. In this case, the zero values were removed from the read counts matrix before the RAS computation, taking into account all the gene profiles of the cells. In Table 2, we reported the new levels of sparsity of the three RAS matrices when the denoising strategies are applied on the read counts matrix. We also reported in brackets the level of sparsity for the essential RAS matrix. We noted a significant decrease in the level of sparsity at the RAS matrix level, using any denoising strategy. More in detail, using MAGIC and SAVER algorithms, almost all zero values were removed from the RAS matrix, and all the cells of all the datasets had no zeros associated with the RASs of essential reactions. The ENHANCE strategy removed most of the zero-values RASs from all the datasets, but a a discrete number of zero values associated with essential reactions was retained. More in detail, $67.9\%$ and $34.01\%$ of the cells for the first and the last dataset, respectively, had at least one zero for a reaction essential for biomass synthesis.

Because denoising was not always sufficient to remove the totality of zero RAS values from essential reactions, we explored the possibility of applying the denoising strategies on the RAS matrix directly, taking into account the RAS profile of the cells. In Table 2, we reported the new levels of sparsity of the three RAS matrices when the denoising strategies are applied on the RAS matrices. Applying denoising before or after the computation of RAS yields quite similar results, for all denoising strategies.

### Denoising enhances correlations in RAS values

To investigate whether the use of a denoising strategy allows uncovering also important reaction-reaction relationships, we analyzed the general level of correlation between the RASs of arbitrary pairs of reactions, as well as the correlation between specific pairs of reactions for which we expected a good correlation.

**Fig. 2:**
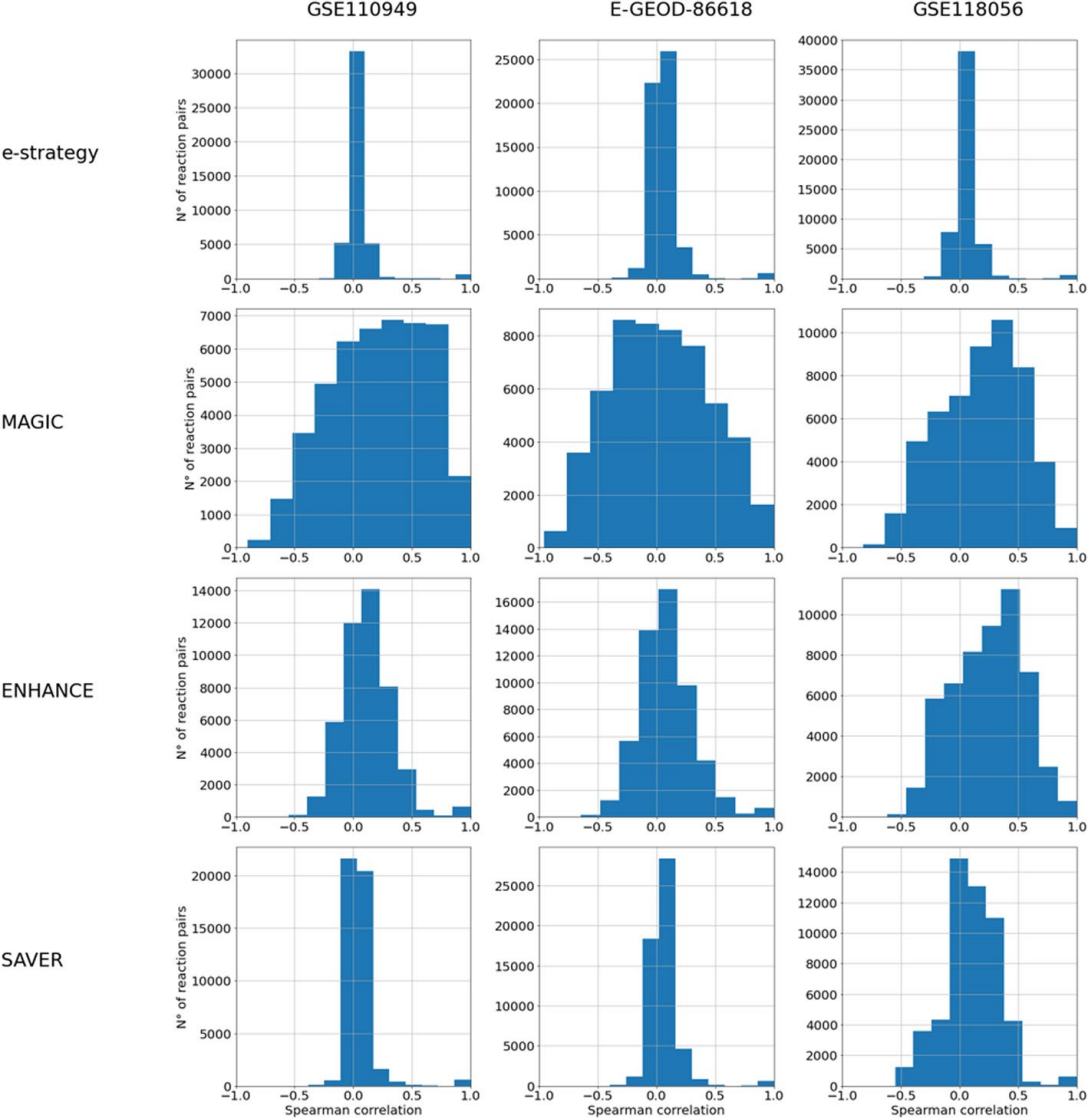
Analysis of the general correlation between RASs. For each dataset and denoising strategies applied on the read counts matrix, we reported the distribution of the pairwise Spearman correlation of the RASs of all the reactions

In Fig. 2, we reported, for each dataset and denoising strategy, applied on the read counts matrix, the distribution of the pairwise correlation of the RASs of all the reactions. It can be noticed a general increase in the level of correlation when applying either MAGIC or ENHANCE, in accordance with the fact that both strategies are based on smoothing the transcriptomics profiles of the cell using the most similar profiles. On the contrary, the SAVER strategy seems to preserve the general low level of correlation between the RASs of all the pairs of reactions. This difference between SAVER and the other denoisers could relate to the extremely sparse expression along with the genes. Indeed, for genes with zero expression value in most cells, the SAVER procedure does not have enough data to base the prediction of the denoised values on [22].

**Fig. 3:**
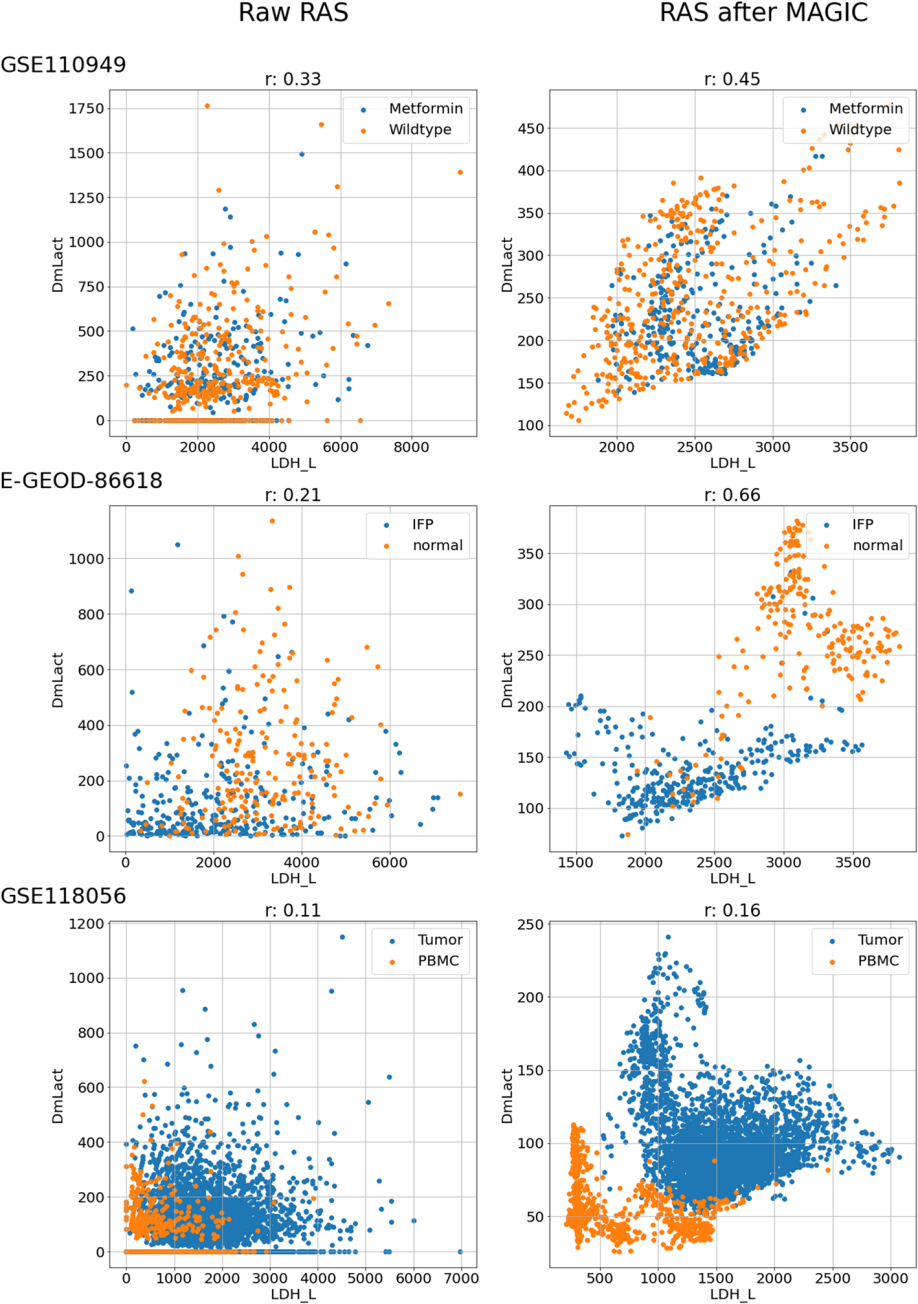
Analysis of the correlation between the RASs of lactate production and secretion reactions. Scatter plots between the RASs of the lactate dehydrogenase (LDH_L) and lactate transporter (DmLact) before (left) and after (right) the application of the MAGIC denoiser on the read counts matrix before RAS computation. Each row represents a different dataset. On the top of the Figures, we reported the value of the Spearman correlation between the two RASs

As a specific example, Fig. 3 illustrates how denoising (MAGIC on the read counts matrix), in this case, is able to recover the important correlation between the RASs of the lactate dehydrogenase (LDH_L) and lactate transporter (DmLact) reactions, for the first and the second dataset. The secretion of lactate - produced by lactate dehydrogenase - outside the producing cells is the final step in the anaerobic utilization of glucose in mammalian cells. Lactate secretion decreases the risk of cell toxicity due to low intracellular pH that would result from intracellular lactate accumulation [23]. The improved correlation between the lactate dehydrogenase and the lactate transporter RASs supports the effectiveness of the denoising procedure in restoring biological significance in scRNA-seq data.

### Denoising enhances flux cluster analysis

To evaluate the efficiency of using the fluxes predicted by FBA as features for cluster analysis, we compared the clustering results with those obtained when using the read counts as features.

**Fig. 4:**
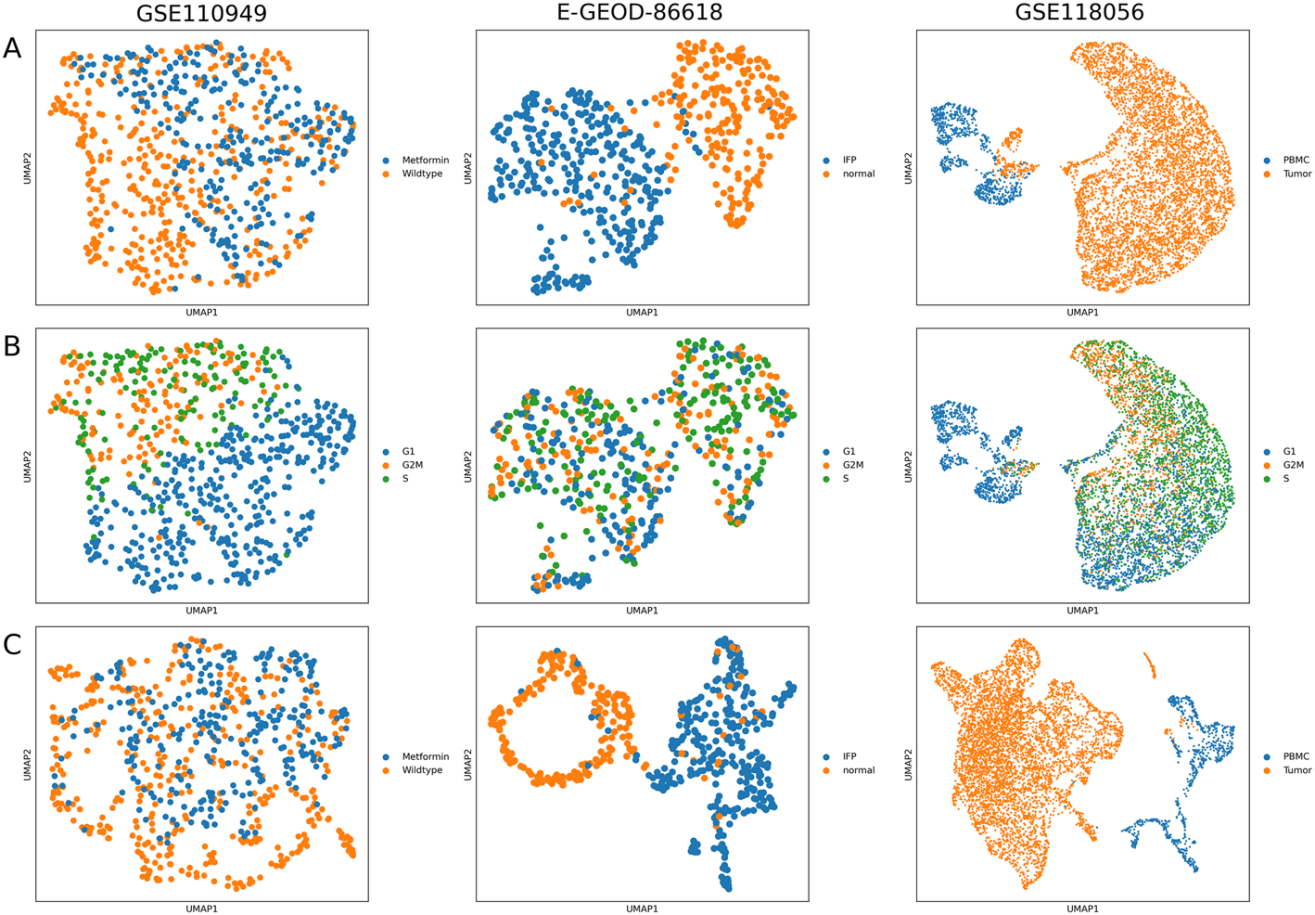
Read counts cluster analysis. Each column represents a different dataset. We reported the UMAPs, where the cells are coloured on the base of the *a priori* knowledge (row **A**) and cell-cycle phase (row **B**). In row **C**, we showed the results of cluster analysis obtained by the application of the MAGIC strategy on the read counts matrix, colouring the cells on the base of the *a priori* knowledge

To this aim, we first performed the cluster analysis on the read counts matrices, and we reported in Fig. 4 the UMAPs of the neighborhood graph of cells, where the cells are coloured on the base of the *a priori* knowledge (Fig. 4A) or cell-cycle phase (Fig. 4B). Each column represents a different dataset. In the first dataset, we noted a moderate separation of the cells between metformin-adaptive and non-adaptive cells. In the second dataset, the major separation between the cells is between normal and IFP cells. Finally, the major separation between the cells of the last dataset is between PBMC and tumour cells. Regarding the cell-cycle phase, we noted that there is a strong separation between cells characterized by a different phase (G2M, S or G1) of the cell cycle for the first and the last dataset. We verified also that the application of the MAGIC denoising on the read counts matrices does not particularly affect the results of the clustering analysis, Fig.4C, for the three datasets, coloured on the base of the *a priori* knowledge.

**Fig. 5:**
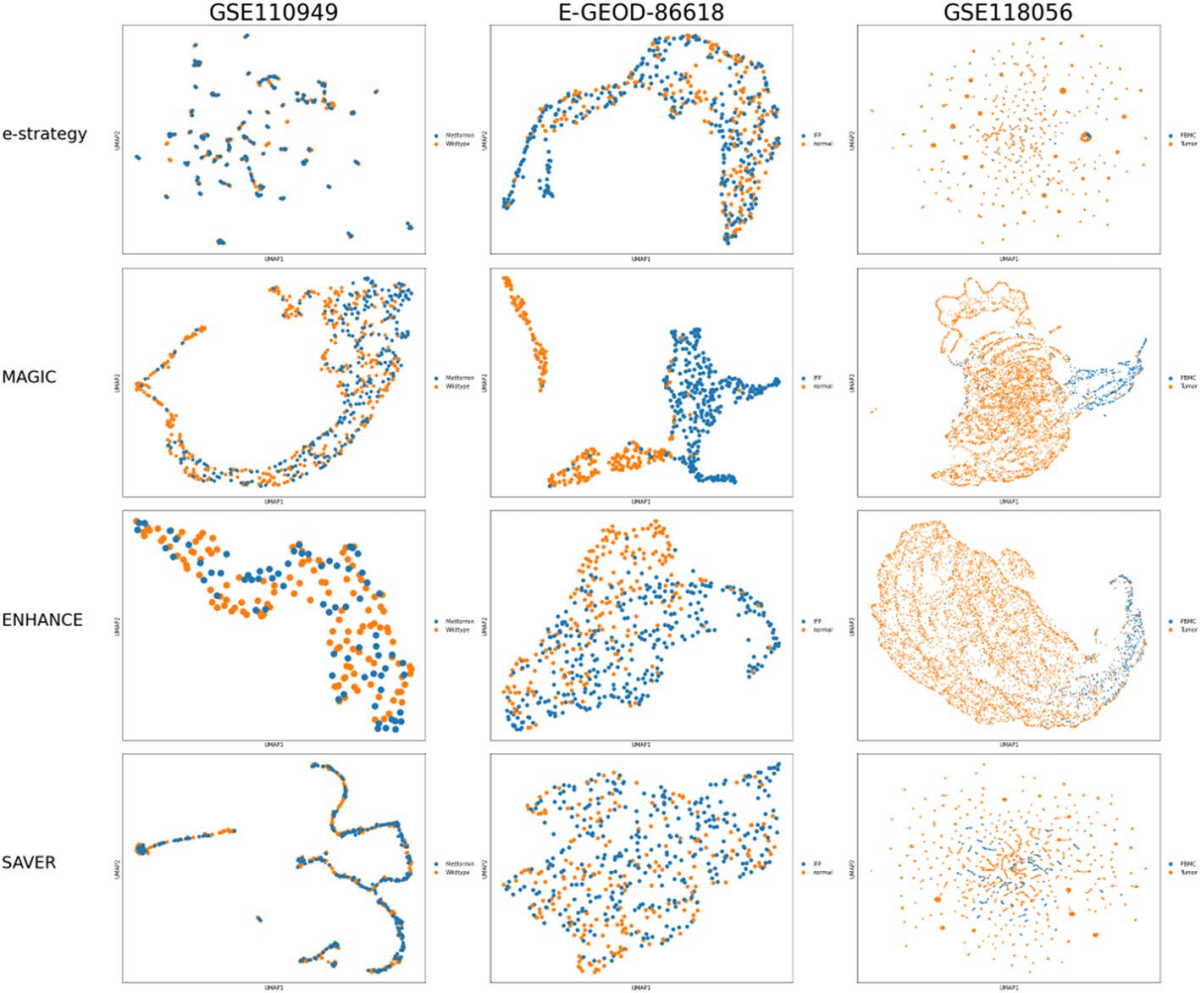
Flux cluster analysis. The results are embedded in a two-dimensional graph using UMAP and coloured using the *a priori* knowledge. Each column represents a different dataset, and each row a different denoising strategy applied on the read counts matrix before the computation of the RAS matrix

To evaluate the clustering when using metabolic fluxes as features, we computed the optimal flux matrices. To this aim, we had to choose a target objective of FBA for Eq. 2. Since the first and the third dataset showed a strong separation between cells in the G1 phase and in the S/G2M phases (see Fig. 4B), we wanted to investigate possible growth-rate differences by setting the biomass synthesis rate as target objective of Eq. 2. For the second dataset, since most cells appear to be non-proliferative, we set instead the ATP maintenance as objective target. The results of cluster analysis performed on the optimal flux matrices are reported in Fig. 5. Each column of Fig. 5 represents a different dataset and each row a different denoising strategy performed on the TPM matrices. In all the UMAPs, we coloured the cells on the base of *a priori* knowledge. We also reported the UMAPs coloured as a function of the cell-cycle phase in Additional file 1: Fig. S1.

From a first qualitative analysis, one can appreciate the substantial difference between using the $\epsilon$-strategy and the MAGIC algorithm. Indeed, it can be observed that the role of the *a priori* knowledge and cell-cycle effect, previously observed in Fig. 4, in determining the cell profiles completely vanished for all the datasets when using the $\epsilon$-strategy (Fig. 5 and Additional file 1: Fig. S1).

On the contrary, MAGIC preserves the good separation between normal and IFP cells for the second dataset and between normal and tumour cells for the third dataset. It is worth noticing that the separation between metformin-adaptive and non-adaptive cells for the first dataset, observed in Fig. 4A, is not preserved equally well, but the cell cycle effect, observed in Fig. 4B, is conserved. The low level of separation between metformin-adaptive and non-adaptive cells on metabolic fluxes be related either to the absence of a strong difference on central carbon metabolism (that is the focus of the ENGRO2 model) or to the moderate level of separation of their transcriptional profiles.

Regarding ENHANCE and SAVER, they preserve quite well the separation between IFP and normal cells for the second dataset, but not as well as MAGIC. Regarding the first dataset, the quality of the UMAP plots of ENHANCE and MAGIC appear comparable, whereas in the SAVER plots, the cells appear sparsely distributed. However, for SAVER, the separation between cells at different cell-cycle phases is preserved (Additional file 1: Fig. S1.). For the last dataset, ENHANCE and SAVER fail to properly separate the cells. A possible explanation of the poor performance of ENHANCE is that we removed most cells from the cluster analysis because they present zero values in at least one essential reaction. The poor performance of SAVER could relate to the presence of genes displaying extremely sparse expression, as already discussed above. Therefore, a stronger quality check on the read counts matrix could improve the performances.

### Denoising enhances the correlation between growth rate and cell cycle phase

In eukaryotic cells, proliferation results from the intersection of two interacting processes: the production of biomass – sometimes referred to as the growth cycle – and the DNA division cycle that accomplishes accurate duplication of the genetic material and its portioning between the two daughter cells. At cell division, a parent cell produces two daughter cells. Each newborn cell grows in size, and after a time corresponding to the time required to double cell mass (MDT), each cell will divide again. Mass accumulation takes place continuously during the cell cycle. Recent studies indicate that the rate of cell mass accumulation is higher for larger cells, approximating - with some deviations - an exponential rate [24]. As a result, the mass accumulation rate (MAR, i.e., the tangent at any given point of the growth curve) is higher in cells in G2M than in cells in S, which have a higher MAR than G1 cells.

**Fig. 6:**
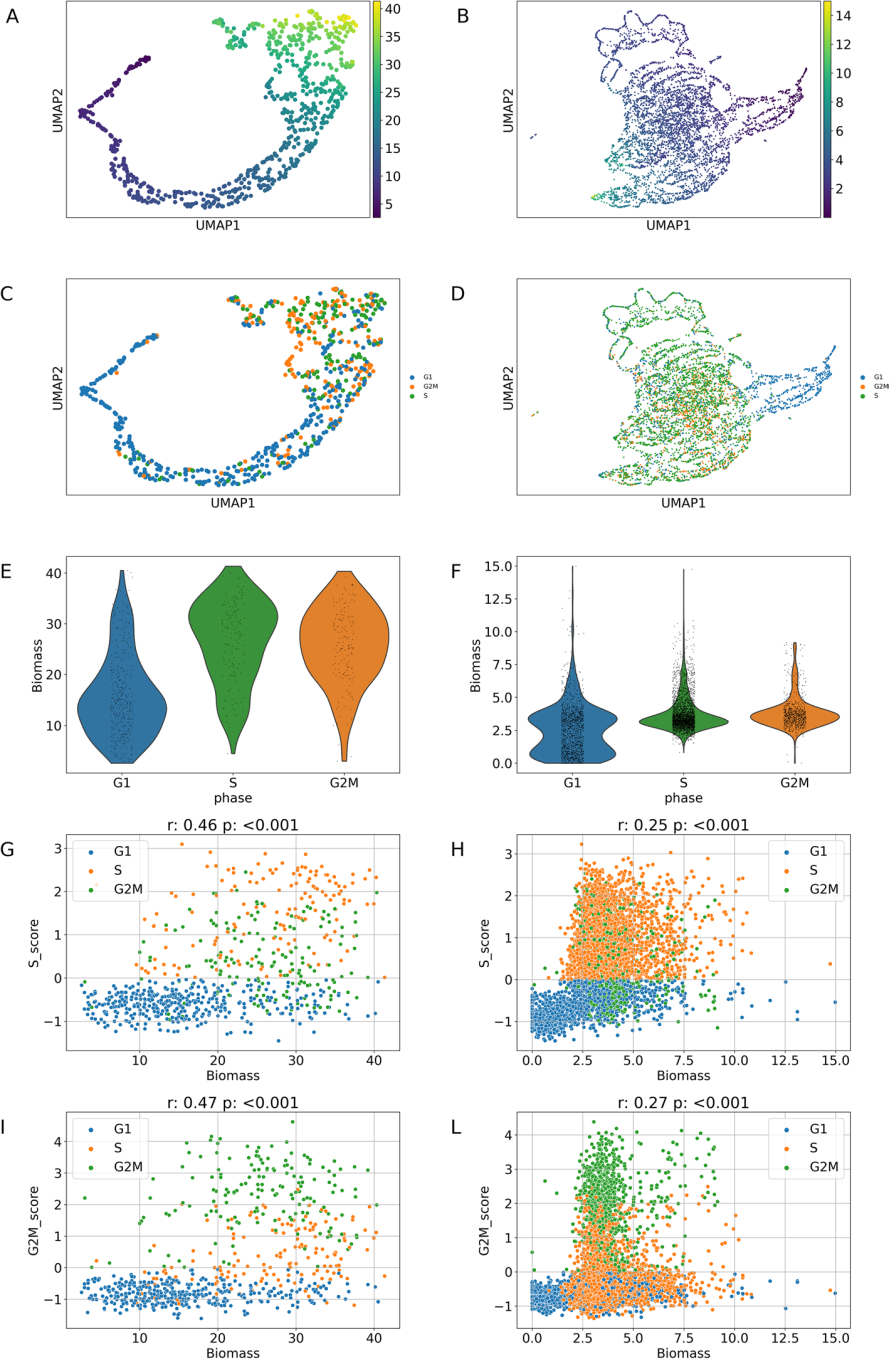
Correlation between biomass synthesis rate and cell-cycle. Summary of the correlation between biomass synthesis rate (Biomass in the Plots) and cell cycle for the first dataset (left column) and third dataset (right column), with MAGIC applied on the read counts matrices. We reported: i) the UMAPs from Fig. 5 coloured as a function of the biomass synthesis rate (A and B) and cell-cycle phase (C and D); ii) the violin plots (E and F) showing the distribution of the cells on the base of biomass synthesis rate and grouped for cell-cycle phase; iii) the scatter plots between the biomass synthesis rate and the S-score (G and H) and G2M-score (I and L). On top of these last Figures, we reported the value of the Spearman correlation with the associated p-value

Hence, we investigated how the use of a denoising strategy affects the possible relationship between biomass synthesis rate and cell cycle phase, for the datasets including proliferative cells (i.e., first and third dataset). Figure 6 shows that denoising (MAGIC, in this case) effectively recovers the correlation between the optimized biomass synthesis calculated by FBA and the cell cycle phase (G1, S, or G2M) deduced from the single-cell expression of signature cell cycle genes.

To further analyze this aspect, we reported the same UMAPs of Fig. 6 but coloured as a function of the biomass synthesis and cell-cycle phase, for the first (Fig. 6A, B) and the third (Fig. 6C, D) dataset. For both datasets, we noticed that the cells in the G2M/S phase show a higher biomass synthesis rate compared to cells in the G1 phase. To better highlight this result, we reported the violin plots (Fig. 6E, F) showing the distribution of the cells on the base of biomass synthesis rate and grouped for cell-cycle phase. Besides, we reported the scatter plots between the biomass synthesis rate and the S-score (Fig. 6G, H) and G2M-score (Fig. 6I, L). We remark that denoising increases the Spearman correlation between biomass synthesis rate and S-score from 0.12 to 0.46 and from 0.15 to 0.25 for the first and the last dataset, respectively. Along similar line, the Spearman correlation between biomass synthesis rate and G2M-score increases from 0.22 to 0.47 and from 0.14 to 0.27 for the first and the last dataset, respectively. All together, these results indicate that the application of denoising improves the prediction of growth rates.

## Discussion

Our preliminary results indicate that the MAGIC algorithm represents a good denoising strategy to remove possible false zero values from the RAS matrix, whereas ENHANCE and SAVER showed some limitations with respect to the quality of the flux cluster analysis (SAVER) and in removing all zero values from the essential reactions (ENHANCE). However, our conclusions might not always hold, because we used the default parameters for each denoiser, while one can expect an improvement of the results if parameters are differently tuned.

Our results also suggest the need to develop denoising strategies specific for the RAS matrix. On the one hand, applying current denoising strategies on the read counts matrix might affect the evaluation of the GPRs since the entries of the read counts matrix are modified based on the comparison of the entire transcriptomics profile of the cells, which involves a large number of genes not directly connected with metabolism. On the other hand, applying current strategies on the RAS matrix rather than on the read counts matrix does not seem to significantly improve results. Moreover, the assumptions of denoising algorithms conceived for read counts, such as the assumption of negative binomial distribution of SAVER, do not necessary hold for RAS values. Hence, as further work, we intend to explore the possibility of incorporating metabolic constraints, such as the correlation between the activity of reactions in a linear chain, into *ad hoc* denoising algorithm.

Remarkably, we observed a good correlation between the optimized biomass synthesis rate calculated by FBA and the cell cycle position deduced from the single-cell expression of signature cell cycle genes. To our knowledge, this is the first time that such correlation has been assessed, and this result may have important implications.

Although recent data correlating metabolism to cell cycle progression are accumulating in a model eukaryotic organism (see [25] for a recent review), more investigation on the subject is required. Cetin et al. [26] recently showed that the mass accumulation rate strongly correlates with therapeutic susceptibility in a panel of human multiple myeloma cell lines. The experimental MAR assay involves using complex microfluidics to actually “weigh” single cells. The availability of a surrogate computational MAR (i.e., the biomass function rate) obtainable from standard single-cell sequencing experiments could thus be a valuable tool in designing personalized drug treatments.

## Conclusion

Stratification of intermingled cell populations, taken, for example, from biopsies, xenografts or organoids, with a focus on metabolic properties, is particularly important to address drug resistance in complex diseases, such as cancer, Parkinson or diabetes, which involve a reprogramming of cell metabolism. In this work, we investigated the possibility of using COBRA methods to simulate numerically single-cell metabolic fluxes integrating single-cell RNA-seq data into constrained-based models. The metabolic fluxes are features that can be used for cluster analysis, aiming at identifying sub-populations of cells within a mixture, sharing distinctive metabolic properties. They might also be combined with other omics to perform multi-omics cluster analysis.

Given that the use of COBRA methods to extract fluxes from bulk transcriptomics data have been widely addressed in the literature, we focused on the pre-processing steps that are specifically required for single-cell data while relying on the most basic COBRA method for feature extraction, that is, FBA.

To this aim, we used three public scRNA-seq datasets, for which we had *a priori* knowledge of the cell mixture composition. As a first analysis, we pointed out the necessity to remove false zero values from the RAS computed from the read counts, in order to constrain and simulate metabolic fluxes. The problem is represented by the presence of reactions that, if constrained to be zero, impair the simulation of flux distributions consistent with expected metabolic function. We remark that this problem impacts a large family of COBRA methods, besides FBA.

As an alternative to the mere removal of constraints from such reactions or their replacement with a small positive value, we here proposed the application of denoising strategies on the read counts matrix. We reported the results of clustering on the three datasets under study and for three state-of-the-art denoising strategies.

We showed that denoising improves: i) the correlation between the expression of reactions within the same linear metabolic pathway, ii) the clustering of cells into expected sub-populations; iii) the correlation between cell cycle phase and biomass synthesis rate.

The application of methods to denoise scRNA-seq data is currently debated because of the possible generation of false-positive values and its non-obvious usefulness for downstream analyses [19]. In fact, we have shown that clustering of single cells is effective also when no denoising is applied. On the contrary, our results indicate that denoising is a fundamental step to integrate scRNA-seq data into FBA simulations.

## Material and methods

### Datasets

In this work, we considered three public scRNA-seq datasets:

- *GSE110949* [27] Composed of 19,826 genes for 2000 MDA-MB-231 breast cancer cells. The dataset was obtained to study the acquired resistance of breast cancer cells to Metformin. MDA-MB-231 cells were maintained as 2D cultures in media containing 0 or 2 mM metformin for more than 8 weeks. Following this period, rates of cell proliferation in 2D cultures were comparable between control (non-adapted) and treated (metformin-adapted) cells. The two groups of cells were then cultured as mammospheres for 8-10 days before undergoing RNA-sequencing.
- *E-GEOD-86618* [28] Composed of 23,909 genes for 540 lung epithelial cells. The dataset was obtained in a study with the aim to identify different lung epithelial cell types and associated biological processes involved in the pathogenesis of Idiopathic Pulmonary Fibrosis (IFP). Lung tissue samples were obtained from three control and six IPF patient samples. Epithelial cells were obtained by cell sorting.
- *GSE118056* [29] Composed of 25,066 genes for 11,071 cells. Such a dataset is based on PBMC and tumour specimen from a human patient with MCPyV associated Merkel cell carcinoma treated with T cell therapy and avelumab (anti-PD-L1), from a time point of late immunotherapy resistance.

The dataset transcripts are identified by Ensembl ID (E-GEOD-86618) or Gene Symbol (GSE110949 and GSE118056). For all these datasets, cluster analysis on the read counts matrix were already performed in the original papers and a strong separation between the transcriptomics profiles of the cells was observed, which is consistent with specific *a priori* knowledge.

### Data pre-processing and cell-cycle scoring

Before the computation of the RAS matrix, some pre-processing operations was necessary on the read counts matrix. More in detail, we filtered out cells with less than 2000 detected genes and genes not expressed in at least 3 cells. Then, we normalized each cell by total counts over all genes so that every cell has the same total count after normalization, that is, the Transcript Per Kilobase Milion (TPM) normalization.

On the transcriptomics data, we estimated the cell cycle phase, using the function *score_genes_cell_cycle* of Scanpy toolkit [30] which, given two lists of genes associated to the S and the G2M phases, respectively, calculates the scores and assigns a cell cycle phase (G1, S or G2M). Such function adds three slots in data: a score for the S phase, a score for the G2M phase, and the predicted cell cycle phase, i.e. G1, S, and G2M.

### Denoising strategies

To mitigate the presence of false zero values in the transcriptomics data, we considered three different denoising methods:

- *MAGIC* [31] is a Python-based algorithm that extracts the true similarity between cells by amplifying biological trends, while simultaneously filtering out spurious correspondences due to noise in the data. First, to overcome the problem of data sparsity, the nearest neighbor graph based on cell-cell expression distance is built. Then, an affinity matrix is defined by applying a Gaussian kernel to the principal components of the graph. Lastly, a diffusion process is applied to the similarity matrix to obtain a smoothed, more faithful affinity matrix. The final imputation involves computing the new expression of each gene as a linear combination of the same expression in similar cells, weighted by the similarity strength obtained in the previous steps.
- *ENHANCE* [32] is a Python-based method that combines Principal Component Analysis (PCA) and cell aggregation using K-nearest neighbors to denoise the observed count matrix. The algorithm can be divided into two main steps. The first one accounts for reducing the bias toward highly expressed genes by aggregating the expression of similar cells based on the distance between their principal component scores. The second phase projects the aggregate matrix on the first k principal components, where k is selected to represent only true biological differences. Lastly, the selected components are used to derive the final denoised matrix.
- *SAVER* [22] is an R-based algorithm that estimates the true gene expression levels by modelling observed counts as a Negative Binomial distribution. More in detail, the technical noise in the gene expression signal is approximated by the Poisson distribution, while the gamma prior accounts for the uncertainty in the true expression. The final recovered expression is a weighted average of the normalized observed counts and the predicted true counts.

For the parameters of the various denoising algorithm, we used the default values and we tested their application both on the read counts matrix and the RAS matrix.

### Metabolic network model

To properly consider the role of denoising in the capability of a simulated metabolic model to achieve a metabolic function, we decided to use the recently published metabolic network model ENGRO2 [9]. ENGRO2 is a constraint-based generic core model of human central carbon and essential amino acids metabolism. It contains 484 reactions, 403 metabolites, and 494 genes and represents a follow-up of the core model of human central metabolism ENGRO1 [33]. 337 model reactions are associated with a gene-protein-reaction (GPR) rule. More in detail, there are 202 single-gene GPRs, 99 OR-expression, 13 AND-expression, and 23 complex rules (i.e., logical expression with both AND or OR operator).

The choice of using a core model is motivated by the likely presence of thermodynamically infeasible loops in genome-wide metabolic network reconstructions, such as the latest Recon3D [34] model, which may camouflage essential reactions. The choice of using a generic model, rather than a specific or multi-cellular model [35], is motivated by the fact that the affiliation of a single-cell to a specific cell population is typically not known *a priori*, hence the need for a cluster analysis.

### In silico growth medium

The availability of nutrients (i.e., the growth medium) significantly affects the metabolic fluxes. Since the exact medium composition is mostly unknown even for common in vitro protocols and in vivo models, we decided to consider a rich in silico medium, as done in [14], where all nutrients for which a transporter exists are made available in an unlimited quantity.

### Flux variability analysis

In order to properly limit the flux of an internal reaction *r*, as a preliminary step, the maximum and minimum flux through such reaction, based on extracellular flux constraints, must be determined specifically for each reaction. To this aim, we performed a Flux Variability Analysis (FVA). FVA [36, 37] is a constraint-based modelling technique aimed at determining the maximal (and minimal) possible flux through any reaction of the model and thus evaluating the cell’s range of metabolic capabilities. FVA solves the following two linear programming optimization problems (one for minimization and one for maximization) for each flux $v_j$ of interest, with $j = 1, \dots , R$:

1 $$\begin{array}{*{20}l} {\max /\min \;v_{j} ,} \hfill \\ {S \cdot \vec{v} = \vec{0},} \hfill \\ {\overrightarrow {{v_{L} }} \le \vec{v} \le \overrightarrow {{v_{U} }} ,} \hfill \\ \end{array}$$

where $\vec {v_L}$ and $\vec {v_U}$ represent the possible bounds used to mimic as closely as possible the biological process in the analysis.

### Flux balance analysis

In order to compute the metabolic fluxes, we used FBA [5], which solves the following linear programming (LP) problem:

2 $$\begin{array}{*{20}l} {\max z = f(\vec{v}),} \hfill \\ {S \cdot \vec{v} = \vec{0},} \hfill \\ {\overrightarrow {{v_{L} }} \le \vec{v} \le \overrightarrow {{v_{U} }} ,} \hfill \\ \end{array}$$

where $f(\vec {v})$ in an objective function. In our work $f(\vec {v})$ is a specific target flux $v_j$ to be maximized. We refer to the set of reactions that, when restricted to zero, causes $v_j$ to be zero as well, as *essential reactions* for $v_j$.

### Reaction activity scores

Starting from a RNA-seq count matrix and a list of *R* reactions involved in a metabolic network, the RAS matrix consists of a $\overline{R} \times C$ matrix, where $\overline{R}\le R$ is the number of reactions associated with a GPR, and *C* is the total number of cells. The entries of the matrix are obtained substituting the mRNA abundances in the corresponding GPRs. Then, the logical expressions are solved by taking the minimum transcript level value when multiple genes are joined by an AND operator, and the sum of their values when multiple genes are joined by an OR operator [33, 38]. More in detail, the RAS related to reactions with the AND operator was computed using the following formula:

3 $$\begin{aligned} RAS_j^c= \min {T^c_{g}:g\in G_j}, \end{aligned}$$

where $T^c_{g}$ is the transcriptional expression level of gene *g* for cell *c*, and $G_j$ is the set of genes that encode the subunits of the enzyme catalyzing reaction *j*. The RAS related to reactions with the OR operator is computed using the following formula:

4 $$\begin{aligned} {RAS^c_j= \sum {T^c_{g}:g\in G_j}.} \end{aligned}$$

In the case of GPRs combining both operators, we respected their standard precedence. In case of missing expression value, referred to as NaN (Not a Number), for a gene joined with an AND operator in a given GPR rule, we decided to solve the rule “A AND NaN” as A. Similarly, for a gene joined with an OR operator in a given GPR rule, we decided to solve the rule “A OR NaN” as A.

### RAS-derived constraints to metabolic fluxes

We created a population of metabolic models, starting from the ENGRO2 metabolic network, and computed, for each cell, specific constraints on internal fluxes following an approach similar to [9, 15]. In a nutshell, for each reaction $j=1,\dots , \overline{R}$ and cell $c=1,\dots , C$, an upper bound $U_j^c$ and a lower bound $L_j^c$ to the flux capacity are defined, based on the following formulas:

5 $$\begin{aligned}&{U_j}^c={F^u}_j \times \frac{{{RAS}_j}^c}{\max _c{{{RAS}_j}^c}}, \end{aligned}$$

6 $$\begin{aligned}&{L_j}^c={F^l}_j \times \frac{{{RAS}_j}^c}{\max _c{{{RAS}_j}^c}}, \end{aligned}$$

where $F_j^u$ and $F_j^l$ represent the maximum and the minimum flux that reaction *j* might carry, obtained by FVA (Eq. 1). Therefore, the possible values of flux v_j_ vary between $F_j^l$ and $F_j^u$. Note that if the RAS value of a reaction is 0 in all the cells for, the corresponding upper bound is kept to 0 for all the cells. Moreover, for reactions not associated with a GPR, the upper and lower bounds correspond to $F^u_j$ and $F^l_j$, respectively.

For a comparison, we considered also the possibility of applying an $\epsilon$-strategy [15]. This strategy is based on the use of the following formulas to integrate the RAS values:

7 $$\begin{aligned}&U_j^c=\epsilon +\left( F_j^u -\epsilon \right) \frac{RAS_j^c}{\max _c{RAS_j^c}}, \end{aligned}$$

8 $$\begin{aligned}&{L_j^c=-\epsilon +\left( F_j^l +\epsilon \right) } \frac{RAS_j^c}{\max _c{RAS_j^c}}, \end{aligned}$$

where the parameter $\epsilon > 0$ is used to mitigate the effects of the false zero values in the RAS matrix. In all the simulations, we set $\epsilon =0.01$. The original application of such strategy [15] also takes into account the bulk profile of the bulk samples, if present, to pre-process the data and remove possible false zero values. However, in our datasets, the bulk profiles are not present, so we cannot apply such pre-processing step.

### Cluster analysis

The final result of the computational steps described above consists in a matrix of single-cell optimal fluxes $f^c_j$, with $j=1,\dots , R$, and $c=1,\dots , C$, which we used to perform cluster analysis.

Cluster analysis on the optimal fluxes $f^c_j$ matrix and on the original read counts matrices were performed using Scanpy toolkit [30]. For the scRNA-seq data, we applied quality check, TPM normalization, $\log (x+1)-$transformation, gene scaling, and dimensional reduction using Principal Component Analysis (PCA). Then, we used the Leiden algorithm [39] to cluster the cells. The numbers of PCs (5, 10, 15, or 20) and the hyper-parameters of the Leiden algorithm was selected as the ones which maximized the Silhouette index for the obtained clusters.

We repeated a similar workflow for the optimal fluxes $f^c_j$ matrix. As quality check operation, we removed all those cells having a zero value for an essential reaction of the objective target yet. Then, we applied reaction scaling, dimensional reduction and the Leiden algorithm to cluster the cells.

## Supplementary Information

**Additional file 1**. Cell-cycle effect on flux cluster analysis.

## Data Availability

All RNA-seq data are available from the Gene Expression Omnibus database (accession numbers GSE110949, GSE118056) or EBI Single Cell Expression Atlas (accession number E-GEOD-86618). For analyzing RNA-seq data and performing single-cell cluster analysis (both on read counts and flux matrices) we used the functions provided by the Scanpy toolkit [30]. For FBA, we used the functions provided by the Cobrapy toolkit [40], and GLPK solver. For applying denoising strategies, we used the codes of the original implementation of the algorithms. For the hyper-parameters of the various denoising algorithms, we used the values provided as default in the original implementations. Scripts to reproduce results are available at https://github.com/CompBtBs/DenoisingFBA
