# Supplementary figures and images for: Combining denoising of RNA-seq data and flux balance analysis for cluster analysis of single cells
Source: BMC Bioinformatics. 2022 Oct 25;23(Suppl 6):445. doi: 10.1186/s12859-022-04967-6 (PMC9597960; doi:10.1186/s12859-022-04967-6)

**Additional Files**

Additional file 1 – Cell-cycle effect on flux cluster analysis

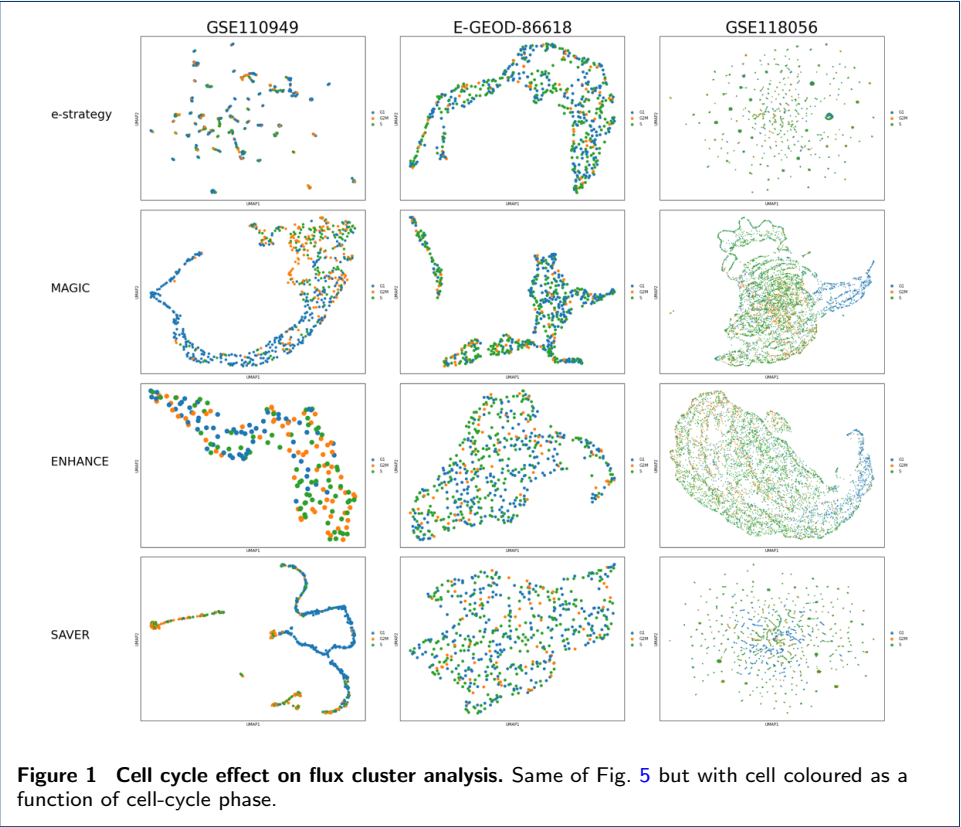

Supplement: Supplementary file 1 — Additional file 1. Cell-cycle effect on flux cluster analysis. [file 12859_2022_4967_MOESM1_ESM.pdf]
